# Supplementary material for: Hyperspectral environmental illumination maps: characterizing directional spectral variation in natural environments
Source: Opt Express. Author manuscript; Available in PMC 2020 Feb 18. (PMC7028397; doi:10.1364/OE.27.032277)
Supplement: Appendix [file EMS85786-supplement-Appendix.pdf]

Optics EXPRESS

Appendix

Figures 8 and 9 show directional spectral variation for other outdoor and indoor scenes, respectively. Here, we see that the degree of spectral variation depends somewhat on the scene. However, we observe a similar general trend as described in the main text. That is, for outdoor scenes as shown in Fig. 8, light from above tends to be more bluish and high luminance whilst lights from below have less energy in the short-wavelength region and lower intensity. Indoor scenes have relatively uniform spectral shape regardless of direction, but regions that include large windows or that are open to the sky have particularly high luminance.

Optics EXPRESS

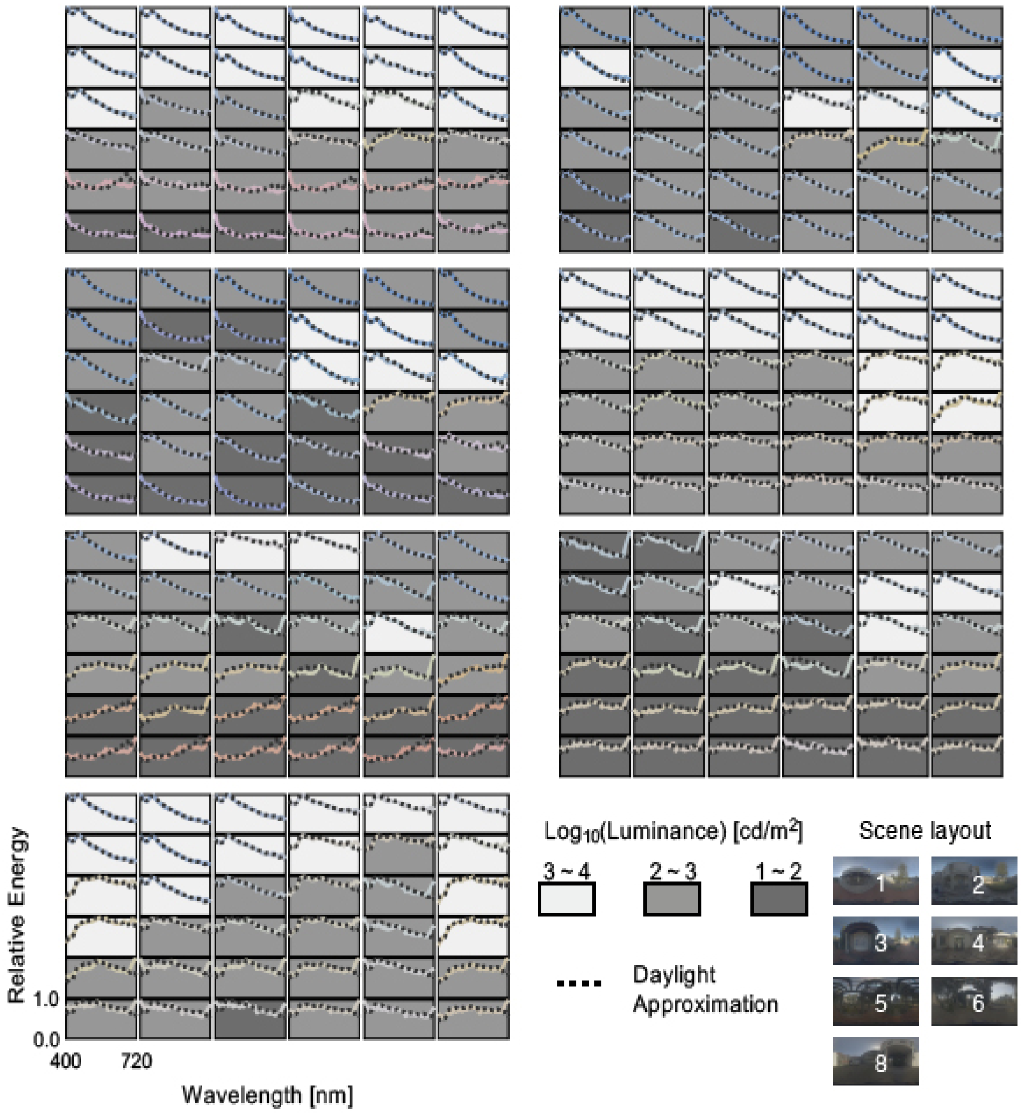

Fig. 8. Directional spectral variation for the remaining 7 outdoor scenes. The way the data is plotted follows Fig. 4. The colour of each curve is the sRGB colour of the spectrum. The background colour represents the log luminance level. The black dotted line indicates the spectrum derived from CIE daylight basis functions.

Optics EXPRESS

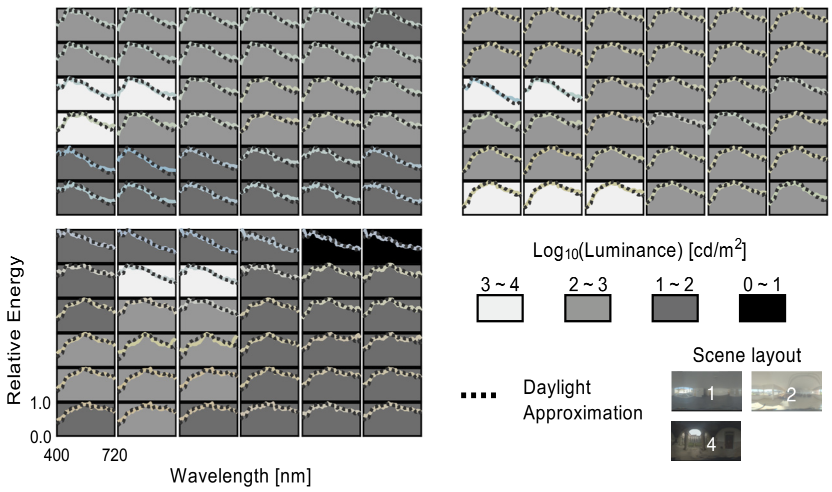

Fig. 9. Directional spectral variation for the remaining 3 indoor scenes. The colour of each curve is the sRGB colour of the spectrum. The background colour represents the log luminance level. The black dotted line indicates the spectrum derived from CIE daylight basis functions.
